# Mutability of Nucleation Particles in Reactive Salt Hydrate Phase Change Materials

**DOI:** 10.1021/acs.jpcc.4c03913

**Published:** 2024-10-07

**Authors:** Denali Ibbotson, Sophia Ahmed, Patrick J. Shamberger

**Affiliations:** † Department of Materials Science and Engineering, Texas A&M University, College Station, Texas 77843, United States

## Abstract

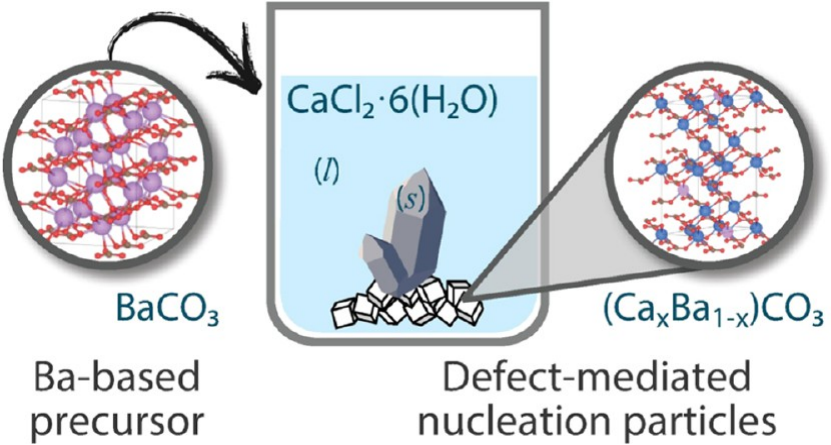

Nucleation particles, solid phases dispersed throughout a medium to decrease the energy barrier for solidification or other reversible phase transitions, are generally selected on the basis of structural or interfacial energy considerations between the host phase and the solid phase that is crystallizing. However, the existence of chemical reactions between the nucleation particles and the host phase can obscure these underlying relationships, thereby complicating the process of selection of active nucleation particle phases. Here, we reveal the origin of nucleation activity of barium-based nucleation particles in the salt hydrate calcium chloride hexahydrate (CCH), a candidate for near room temperature thermal energy storage. We demonstrate that these compounds undergo a series of cation exchange and secondary precipitation reactions, resulting in an assemblage of solid precipitates with some degree of limited solid solution, which collectively dramatically reduce undercooling in CCH, but which obscure the identification of a single crystalline phase primarily responsible for the nucleation of crystalline CCH from the liquid. Importantly, this result illustrates a pathway to harness in situ chemical reactions to generate stable active nucleation particles in reactive phase change materials, which may not be readily synthesized by alternative methods, or which may not be active or remain stable when added in isolation.

## 1 Introduction

Salt hydrate systems are a class of phase change materials (PCMs) with great potential for use in thermal energy storage (TES) applications due to their low cost, high gravimetric and volumetric energy density,^1,2^ and the ability to exploit salt hydrate eutectics to define bespoke invariant eutectic temperatures to meet application-specific requirements.^3^ Despite these advantages, salt hydrates are susceptible to significant degrees of undercooling, a nucleation-limited phenomena where a liquid cools to a metastable temperature without solidifying. Examples of stoichiometric salt hydrates which exhibit significant undercooling include magnesium chloride hexahydrate,^2,4^ calcium chloride hexahydrate,^5,6^ lithium nitrate trihydrate,^7^ sodium sulfate decahydrate,^8^ and magnesium nitrate hexahydrate.^2^ Strategies to reduce undercooling generally consist of either passive approaches including introducing solid particles or other defects which serve to stabilize incipient crystal nuclei, or active approaches, which include introducing pressure or electric field fluctuations to overcome energetic barriers to nucleation. Solid surfaces can be introduced by adding discrete nucleation particles,^8,9^ by adding a matrix which serves to stabilize shape and also nucleate solids,^10^ or by adding a thickener or polymeric gel, which can induce nucleation by molecular-scale interactions.^11^ Active methods which have demonstrated some success include physical agitation,^12^ ultrasonic vibration,^13^ or stimulation by electrical currents.^14,15^ The use of passive methods is often preferred as it eliminates additional system complexity and can promote nucleation of a specific solid phase and suppress the formation of the undesired phase. However, the identification of effective nucleation particles for a specific system remains a challenge as strategies to identify effective nucleation particles on an ad hoc basis are not universally successful, nor do these strategies universally explain the efficacy of certain existing nucleation particles. This challenge is exacerbated in systems where nucleation particles undergo chemical reactions with host PCMs.

The use of nucleation particles (NPs) to promote nucleation within a salt hydrate system has a rich history, dating from work in the 1950s on seeding of water ice crystals,^16^ which was subsequently extended to salt hydrate systems by Telkes and others.^8^ Since this point, most emphasis has been placed on different lattice-matching structural relationships between the introduced solid surface and the crystallizing phase, including either isostructural or epitaxial relationships. The efficacy of lattice-matched nucleation surfaces is attributed to a reduction in the difference of interfacial energy between the solid–solid interface (*i*.*e*., between the nucleating surface and the crystallizing phase) which serves to minimize the energetic barrier to nucleation.^16^ Isostructural (synonymous to isotypic) nucleating surfaces are phases that have (1) the same space group, (2) comparable atomic positions, although different chemistries, and (3) similar lattice parameters as the crystallizing phase.^17^ As a prime example, in Glauber’s salt (sodium sulfate decahydrate) it was observed that borax (sodium tetraborate decahydrate), a material with a <15% disagreement between the crystal lattice parameters, is an effective isostructural phase which is only weakly soluble in saturated sodium sulfate solutions, and is capable of nucleating sodium sulfate decahydrate.^8^ In contrast, epitaxial nucleating surfaces are phases that have (1) at least one crystallographic plane with similar symmetry and lattice parameters as a plane in the crystallizing phase, and (2) comparable atomic positions, although potentially different chemistries.^9,18,19^ As an example, for lithium nitrate trihydrate, it has been experimentally validated that an epitaxially matched NP, likasite, was effective in reducing the undercooling in the system.^9^ In another study, it was found that several cubic carbide and nitride compounds were effective nucleators for gallium, due to their quasi-epitaxial relationship with the (010) plane of crystalline gallium, where the lattice parameters are similar but the atomic positions deviate significantly.^19^

In general, the use of structurally similar NPs (sharing either isostructural or epitaxial relationships with the host PCM) is conditioned on the premise that these particles will retain their structure in the presence of the specific PCM. However, this may not be the case, particularly in the case of reactive or corrosive PCM phases. Thus, this strategy is subject to both false positives, where the existence of a lattice coherence has little to no demonstrable effect on nucleation rates in a PCM, as well as false negatives, where the effective NPs have no apparent structural relationship with the solidifying phase. In both cases, interpretations may be complicated because nucleation is an interfacially mediated property and thus, is sensitive to the nature of the surface of the NP. For example, the surface of solid NPs can restructure when exposed to fluids due to redox reactions, ion-exchange reactions, intercalation of water or ions, or other reactive mechanisms. Therefore, the assumption that NP surfaces will retain the crystal structure of the solid NP when they are introduced into a reactive salt hydrate system may not be valid. Thus, this mechanism will need to be considered when trying to understand why a particular NP will actively nucleate a phase and when defining a strategy to select an active NP for a specific solidifying phase.

Calcium chloride hexahydrate (CCH; CaCl_2_·6[H_2_O]) is an attractive salt hydrate for thermal energy storage applications due to its high energy density (170–190 J·g^–1^), its low melting temperature (29.8 °C), and its low cost (<0.3 US$·kg^–1^).^2,5,20,21^ However, calcium chloride hexahydrate can experience a metastable state, wherein it remains in a liquid state below the equilibrium melting point, and thus CCH can experience undercooling, which can lead to the eventual degradation of the salt hydrate and can hinder its utilization as a PCM in TES. Undercooling in pure calcium chloride hexahydrate has been observed to range from 10–25 °C,^5^ where undercooling was measured with an 80 g sample that was heated to at least 10 °C above its melting point before being cooled to room temperature. In a different study, undercooling in CCH was measured using a differential scanning calorimeter, with a cooling rate of 1 °C·min^–1^ and measured undercooling up to 15.5 °C.^21^ To address this issue, NPs are often added to the system to reduce the undercooling. Literature has reported the previous use of NPs including barium carbonate, barium hydroxide, strontium carbonate, strontium chloride hexahydrate, and several others (Table 1) to be effective in reducing the undercooling of calcium chloride hexahydrate. As an example, strontium chloride hexahydrate is isostructural to calcium chloride hexahydrate, sharing the same space group and similar lattice parameters, which is often the justification for its utilization. However, strontium-based nucleation particles are at least partially soluble in calcium chloride hexahydrate under most operable conditions, introducing some uncertainty about its long-term efficacy as a nucleation particle.^22^

**Table 1:** Overview of Literature Reported Nucleation Particles

nucleation particle	space group	wt % added	DT (°C)^a^	refs
pure PCM	*P*3_2_1 (No. 150)		22.5	(5)
CaBr_2_·6(H_2_O)	*P*3_2_1 (No. 150)	0.5	17.4	(5)
BaO	*Fm*3̅*m* (No. 225)	0.1	0.3	(5)
BaF_2_	*Fm*3̅*m* (No. 225)			(5)
BaCl_2_	*Pnma* (No. 62)	0.5	0.0	(5)
BaI_2_	*Pnma* (No. 62)			(2)
BaCO_3_	*Pmcn* (No. 62)	0.5	0.0	(5)
Ba(OH)_2_	*P*2_1_/*n*(No. 14)	0.5	0.0	(5)
Ba(NO_3_)_2_	*Pa̅*3 (No. 205)			(2)
BaSO_4_	*Pnma* (No. 62)	0.1	0.2	(5)
BaS_2_O_3_	b			(2)
BaCl_2_·2(H_2_O)	*P*2_1_/*n* (No. 14)			(5)
BaI_2_·6(H_2_O)	c	0.5	0.0	(5)
Ba(OH)_2_·8(H_2_O)	*P*2_1_/*n* (No. 14)			(23)
SrF_2_	*Fm*3̅*m* (No. 225)			(5)
SrCO_3_	*Pmcn* (No. 62)			(2)
Sr(OH)_2_·8(H_2_O)	*P*4/*ncc* (No. 130)			(2,24)
SrCl_2_·6(H_2_O)	*P*3_2_1 (No. 150)	0.5	6.2	(5)
SrCl_2_·6(H_2_O)	*P*3_2_1 (No. 150)	1.0	1.8^d^	(21)
SrBr_2_·6(H_2_O)	*P*3_2_1 (No. 150)	0.5	14.0	(5)
SrI_2_·6(H_2_O)	hexagonal	0.5	12.4	(5)
NiCl_2_	*R̅*3*m* (No. 166)			(2)

a Measured by mL-scale “freeze tests”, except where noted. Sample of 10–100 g of PCM (with and without added nucleation particles), was heated to above its melting point and then cooled while measuring the time–temperature results.^5^

b No space group defined, however is claimed to be nonisostructural to CaCl_2_·6(H_2_O).^2^

c No space group defined, but was noted to crystallize in the hexagonal system and claimed to be isomorphous to CaCl_2_·6(H_2_O).^5^

d Measured by μL-scale “DSC tests”. Sample of <10 mg cooled at a rate of 1 °C·min^–1^ under an inert (nitrogen) atmosphere.

Portions of this table are adapted with permission from ref (5). Copyright [1992] [Elsevier].

In this study, we evaluated potential NPs for calcium chloride hexahydrate, the most effective of which contained barium, were relatively insoluble in water, and did not have any apparent structural relationship to CCH. However, it was observed that upon introduction to liquid CCH, the barium-based nucleation particles undergo a cation exchange reaction resulting in a calcium-dominated solid remnant with some limited Ba solid solution, and multiple secondary reaction phases occur as a consequence of this chemical reaction. Furthermore, it was observed that, taken in isolation, calcium-based solid remnants each individually have little effect on the efficacy of barium-based nucleation particles. Given the lack of structural relationship, we hypothesize that the efficacy of barium-based nucleation particles in reducing undercooling in CCH is potentially due not to underlying lattice or structural relationships, but rather to the existence of atomic-scale defects on the surface of a solid solution phase. Thus, this study reveals the role of cation solubility in ionic compounds on their nucleation activity, and the utility of a strategy based on reactive precursors which result in active solid nucleation particles.

## 2 Methods

### 2.1 Materials

Calcium chloride hexahydrate (CCH; CaCl_2_·6(H_2_O)) was obtained from Sigma-Aldrich, with mass fraction *w* > 0.99 purity (calculated based on dry substance) and was used as received. Table 2 shows an overview of the different NPs that were tested with CCH. All NPs were evaluated by XRD for phase purity and were used as-received.

**Table 2:** Chemical Compounds Evaluated for Nucleation Potency in Calcium Chloride Hexahydrate

chemical compound	nominal composition	chemical purity^a^	space group		supplier
calcium carbonate	CaCO_3_	0.98	*R*3̅*c*	(No. 167)	VWR
calcium hydroxide	Ca(OH)_2_	0.98	*P*3̅*m*1	(No. 164)	Acros Organics
barium oxide	BaO	0.97	*Fm*3̅*m*	(No. 225)	Sigma-Aldrich
barium bromide	BaBr_2_	0.99	*Pnma*	(No. 62)	Thermo Scientific
barium carbonate	BaCO_3_	0.9998	*Pmcn*	(No. 62)	Sigma-Aldrich
barium hydroxide	Ba(OH)_2_	0.95	*P*2_1_/*n*	(No. 14)	Beantown Chemical
barium chloride dihydrate	BaCl_2_·2(H_2_O)	0.99	*P*2_1_/*n*	(No. 14)	Beantown Chemical
barium iodide dihydrate	BaI_2_·2(H_2_O)	0.99	*C*1_2_/*c*1	(No. 15)	Sigma-Aldrich
barium hydroxide octahydrate	Ba(OH)_2_·8(H_2_O)	0.98	*P*2_1_/*n*	(No. 14)	Acros Organics
strontium carbonate	SrCO_3_	0.975	*Pmcn*	(No. 62)	Alfa Aesar
strontium chloride hexahydrate	SrCl_2_·6(H_2_O)	0.99–1.03 ACS	*P*3_2_1	(No. 150)	Beantown Chemical
magnesium carbonate	MgCO_3_	b	*R*3̅*c*/*H*	(No. 167)	Millipore Sigma

a Chemical purity reported on a metals basis.

b Certified reference material pharmaceutical secondary standard.

### 2.2 Experimental Section

#### 2.2.1 Calorimetry

To analyze the effectiveness of different NPs, 2 wt % of different solid compounds (Table 2) were mixed with stoichiometric calcium chloride hexahydrate before analysis. Once the nucleation particle had been in contact with the salt hydrate system for at least 12 h and with a maximum time of 96 h, multiple samples (5–10 mg) were prepared to assess the melting temperature (*T*_fus_) and the heat of fusion (Δ*H*_fus_) of the phase change. All samples were sealed in Tzero aluminum pans with hermetic lids (TA Instruments) to mitigate water loss or absorption to the environment.

A TA Instruments Q2000 differential scanning calorimeter (DSC) was used to determine *T*_fus_ and Δ*H*_fus_ of the different samples to evaluate nucleation particle efficacy. The Q2000 calorimeter was calibrated with a pure indium standard (*w* = 0.9999), using reference values of *T*_fus_ = 156.598 °C and Δ*H*_fus_ = 28.662 J·g^–1^, and with a pure tin standard (*w* = 0.9999), with reference values of *T*_fus_ = 231.9 °C and Δ*H*_fus_ = 60.216 J·g^–1^. The predicted relative uncertainties (*u*_r_) for individual measurements at a 95% confidence interval, is based on repeated analysis of the previously mentioned standards for the Q2000 are *u*_r_(*T*_fus_) = ± 0.0027 and *u*_r_(Δ*H*_fus_) = ± 0.0095 for indium, and *u*_r_(*T*_fus_) = ± 0.00017 and *u*_r_(Δ*H*_fus_) = ± 0.0023 for tin. Any deviations between reported sample averages and references values for the Q2000, , are within ±0.0027 and ±0.0095 for *T*_fus_ and Δ*H*_fus_, respectively. All samples were subjected to heating and cooling cycles at a rate of 10 °C·min^–1^ over the range *T*_fus_ ± 50 °C with a continuous nitrogen flow of 50 cm^3^·min^–1^. Following the standard DSC techniques (ASTM E793–06), the melting peak onset was reported as *T*_fus_, and is defined by the intercept of a linear baseline with the maximum slope tangent to the melting curve. Undercooling (Δ*T*) is defined as the difference between the melting temperature and the solidification (crystallization) temperature (Δ*T* = *T*_fus_ – *T*_crys_), with *T*_crys_ defined by the onset of the abrupt exothermic crystallization peak. Δ*H*_fus_, the heat of fusion, is determined through the integration of the melting peak.

#### 2.2.2 X-ray Diffraction

To identify potential secondary phases in samples, several of the samples were sent to the rapid access 11-BM beamline at the Advanced Photon Source at Argonne National Laboratory. High resolution synchrotron powder diffraction data was collected using beamline 11-BM where the average wavelength used was 0.45971 Å. Discrete detectors covering an angular range from −6 to 28° 2θ are scanned over a 34° 2θ range, with data points collected every 0.001° 2θ and scan speed of 0.01 degree·s^–1^. The 11-BM instrument utilizes X-ray optics with two platinum-striped mirrors and a double-crystal Si(111) monochromator, where the second crystal has an adjustable sagittal bend.^25^ The ion chambers monitor incident flux. A vertical Huber 480 goniometer, equipped with a Heidenhain encoder, positions an analyzer system comprised of 12 perfect Si(111) analyzers and 12 Oxford-Danfysik LaCl_3_ scintillators, with a spacing of 2° 2θ.^26^ Analyzer orientation can be adjusted individually on two axes. A three-axis translation stage holds the sample mounting and allows it to be spun, typically at ∼5400 rpm (90 Hz). A Mitsubishi robotic arm is used to mount and dismount samples on the diffractometer.^27^

The diffractometer is controlled via EPICS.^28^ Data is collected while continually scanning the diffractometer 2θ arm. A mixture of NIST standard reference materials, Si (SRM 640c) and Al_2_O_3_ (SRM 676) is used to calibrate the instrument, where the Si lattice constant determines the wavelength for each detector. Corrections are applied for detector sensitivity, 2θ offset, small differences in wavelength between detectors, and the source intensity, as noted by the ion chamber before merging the data into a single set of intensities evenly spaced in 2θ. The resulting XRD spectra, from the 11-BM beamline, were refined using GSAS-II.^29^

#### 2.2.3 Scanning Electron Microscopy/Energy Dispersive X-ray Spectroscopy

To identify and quantify the elemental composition of the dried residual particulate, scanning electron microscopy (SEM) and energy dispersive X-ray spectroscopy (EDS) were used. Samples were first sputter-coated with 5 nm of platinum/palladium alloy to prevent charging of the samples before SEM imaging (FE-SEM, JSM-7500F, JEOL, JEOL; Tokyo, Japan). SEM images were collected with an acceleration voltage of 20 kV and a working distance of 8 mm. EDS data was collected using INCA software (Oxford Instruments) and the data was fitted with Gaussian peak profiles and converted to atomic percent using copper as the calibration element.

## 3 Results and Discussion

### 3.1 Assessing Efficacy of Nucleation Particles

**Figure 1:**
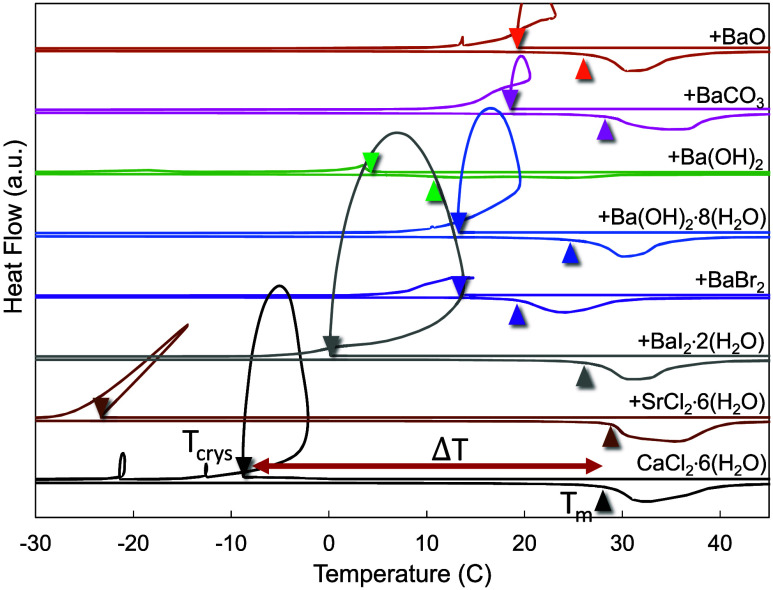
DSC of previously reported nucleation particles with calcium chloride hexahydrate. All samples were of ∼5–10 mg of CCH with ∼2–5 wt % of nucleation particles added and were tested at heating and cooling rates of 10 °C·min^–1^.

Melting characteristics and thermal properties of calcium chloride hexahydrate with different nucleation particles added can be observed in Table 3 and Figure 1 below. The efficacy of NPs to nucleate CCH is defined by their ability to reduce undercooling, which is defined as the difference between the melting temperature and the solidification temperature. It was noted that of the different NPs reported in literature and evaluated here, candidate phases that contained Ba were generally more effective than the NPs that did not contain Ba (*e*.*g*., SrCO_3_, SrCl_2_·6(H_2_O), CaCO_3_), and that this effectiveness spanned across multiple different Ba-containing crystallographic phases (Figure 1). Additionally, it was also observed that several candidate NPs, including strontium chloride hexahydrate, strontium carbonate, and calcium hydroxide, were ineffective in our testing despite being previously reported as effective nucleators.^2,30^ Several different phyllosilicates (talc, montmorillonite, muscovite) were also evaluated as nucleation particles due to their effectiveness in other salt hydrate systems, however these compounds had little to no effect on the undercooling in CCH and thus were not pursued further.^3^ Thus, we conclude that the presence of barium is integral to a reduction in undercooling, as multiple different barium-based nucleation particles reduced the undercooling of CCH, independent of the particular crystal symmetry and lattice parameters of those particular phases.

**Table 3:** Literature Reported Nucleation Particle Crystallographic Overview

		lattice parameters		
nucleation particle	space group	[*a*, *b*, *c*] (Å)	[α, β, γ] (deg)	ref
neat CCH	*P*3_2_1 (No. 150)	[7.876, 7.876, 3.954]	[90, 90, 120]	(31)
CaCO_3_	*R*3̅*c* (No. 167)	[4.99, 4.99, 17.07]	[90, 90, 120]	(32)
Ca(OH)_2_	*P*3̅*m*1 (No. 164)	[3.59, 3.59, 4.90]	[90, 90, 120]	(33)
BaO	*Fm*3̅*m* (No. 225)	[5.51, 5.51, 5.51]	[90, 90, 90]	(34)
BaBr_2_	*Pnma* (No. 62)	[8.28, 4.96, 9.92]	[90, 90, 90]	(35)
BaCO_3_	*Pmcn* (No. 62)	[5.31, 8.89, 6.42]	[90, 90, 90]	(36)
Ba(OH)_2_	*P*2_1_/*n* (No. 14)	[9.41, 7.92, 6.77]	[90, 95.81, 90]	(37)
BaCl_2_·2(H_2_O)	*P*2_1_/*n* (No. 14)	[6.72, 10.91, 7.13]	[90, 91.10, 90]	(38)
BaI_2_·2(H_2_O)	*C*1_2_/*c*1 (No. 15)	[11.042, 7.574, 8.622]	[90, 113.17, 90]	(39)
Ba(OH)_2_·8(H_2_O)	*P*2_1_/*n* (No. 14)	[9.35, 9.28, 11.87]	[90, 99, 90]	(40)
SrCO_3_	*Pmcn* (No. 62)	[5.09, 8.36, 5.99]	[90, 90, 90]	(36)
SrCl_2_·6(H_2_O)	*P*3_2_1 (No. 150)	[7.94, 7.94, 4.11]	[90, 90, 120]	(31)
MgCO_3_	*R*3̅*c*/*H* (No. 167)	[4.63, 4.63, 15.02]	[90, 90, 120]	(32)

### 3.2 Effect of Residual Solids

**Figure 2:**
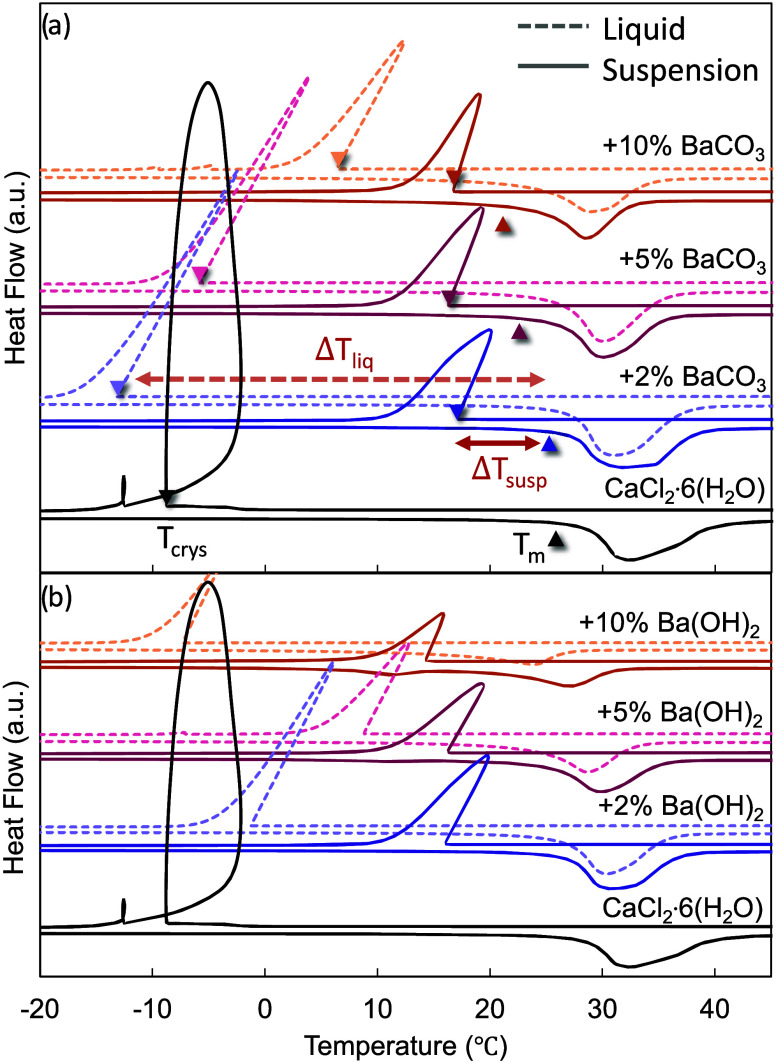
DSC graphs of calcium chloride hexahydrate samples, both with the suspended particles of the insoluble NPs (solid lines) and without, containing only the liquid supernatant (dashed lines), (a) BaCO_3_ in CCH, (b) Ba(OH)_2_ in CCH. *T*_m_ is defined as the onset of melting and is indicated on the graph by an upward pointing arrow; *T*_crys_ is defined as the onset of the nucleation event, in the graph it is indicated by a downward pointing arrow. Δ*T* = *T*_m_ – *T*_crys_ is indicated by the double-headed arrows. Δ*T*_liq_ refers to the undercooling in the case of the sample only containing the liquid supernatant and Δ*T*_susp_ refers to the undercooling when the sample also contains the suspended particles of the insoluble NPs.

Among the effective barium-based nucleation particles, the presence of residual solid phases was critical to promote nucleation in CCH (Figure 2). Most of the barium-based NPs were observed to be weakly soluble in liquid CCH, based on changes in the character of the melting peak. NPs were agitated in liquid calcium chloride hexahydrate and held at an elevated temperature of 50 °C for at least 24 h, after which solid particulates remained at the bottom of the vials even in concentrations as low as 2 wt %. We performed DSC and evaluated undercooling in samples that either included suspended solid particles (solid lines, Figure 2), or included only the liquid supernatant after solid particles gravitationally settled (dashed lines, Figure 2). In each case, the sample was prepared in a hermetically sealed aluminum DSC pan, and was heated and cooled at a rate of 10 °C·min^–1^ between the temperatures of −50 and 80 °C. The melting temperature, *T*_m_, is defined as the onset of the melting endotherm, and the crystallization temperature, *T*_crys_, is defined as the onset of the exothermic nucleation event. The reported undercooling is the difference between the melting temperature and the crystallization temperature, Δ*T* = *T*_m_ – *T*_crys_. It was observed that the inclusion of solid particles substantially reduced undercooling, compared against the samples that contained only the liquid supernatant (Figure 2). Additionally, higher concentration of NPs added to CCH tended to both decrease the melting temperature and broaden the melting peak. This suggests that while the NPs are weakly soluble, the inclusion of residual solid particles is necessary for the reduction of the undercooling.

### 3.3 Evaluation of Isostructural Compounds

Nucleation particles isostructural to a barium-based NP were observed to be ineffective nucleators. To isolate and evaluate the role of crystal structure, strontium carbonate (SrCO_3_), which is isostructural to barium carbonate (BaCO_3_; Table 3), with 4.4% smaller lattice parameters. In contrast to BaCO_3_, which significantly reduced undercooling (Figure 1), SrCO_3_ was ineffective in reducing the undercooling on calcium chloride hexahydrate, despite these two phases being isostructural to one another. Furthermore, among the different effective barium-based nucleation particles, many of them have dramatically different crystal structures and symmetries (as seen in Table 3). Thus, it appears unlikely that a purely structural description of the solid phases added to CCH is sufficient to explain their efficacy at nucleating solid CCH.

### 3.4 Reactive Nucleation Particles

**Figure 3:**
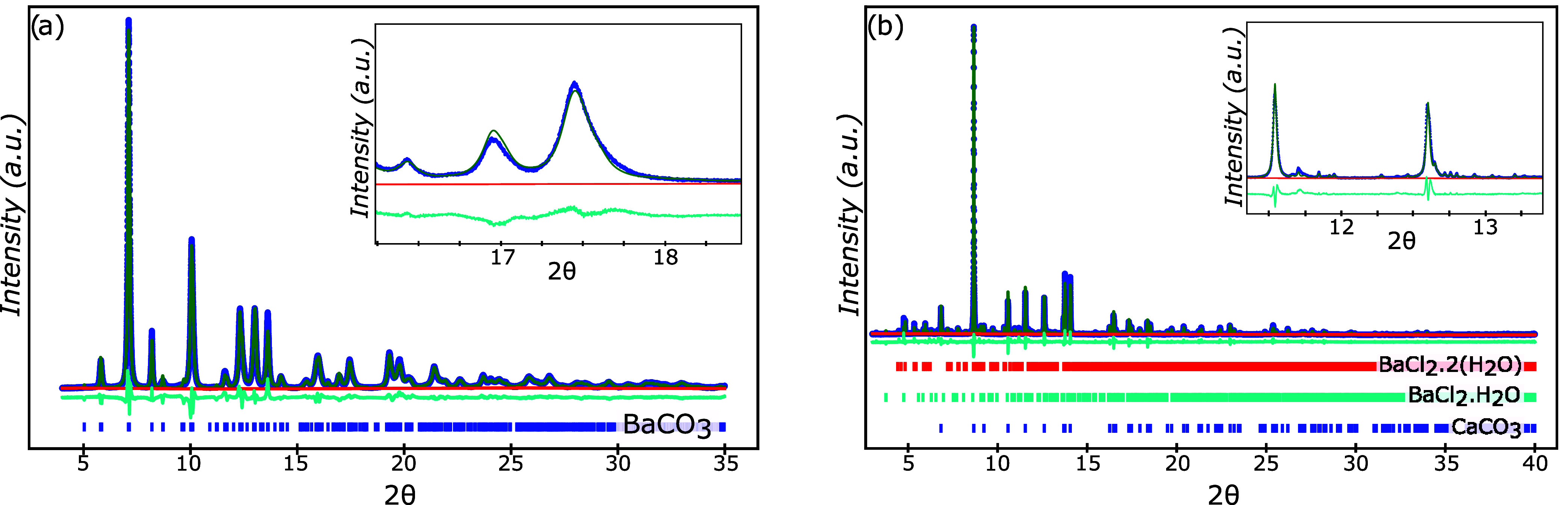
Refined powder diffraction data for (a) as-received barium carbonate (100 wt % BaCO_3_), and (b) for reacted barium carbonate (BaCO_3_), that matches well to the calculated powder diffraction patterns for calcium carbonate (94.7 wt % CaCO_3_), barium chloride dihydrate (3.8 wt % BaCl_2_·2(H_2_O)), and barium chloride monohydrate (1.1 wt % BaCl_2_·(H_2_O)).

**Figure 4:**
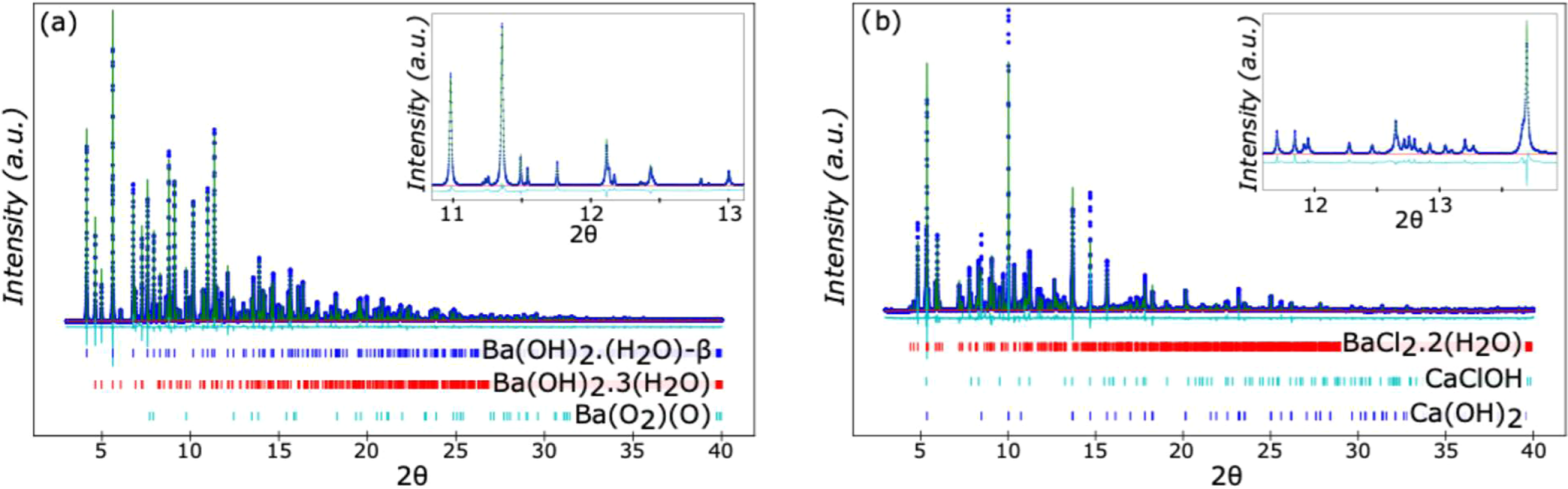
Refined powder diffraction data for (a) as-received barium hydroxide, Ba(OH)_2_, that matches well to the calculated powder diffraction patterns for β-barium hydroxide monohydrate (79.6 wt % β-Ba(OH)_2_·(H_2_O)), barium hydroxide trihydrate (19.1 wt % Ba(OH)_2_·3(H_2_O)), and barium peroxide (1.3 wt % BaO_(2−δ)_), and (b) reacted barium hydroxide (Ba(OH)_2_), that matches well to the calculated powder diffraction patterns for calcium hydroxide (36.3 wt % Ca(OH)_2_), barium chloride dihydrate (29.7 wt % BaCl_2_·2(H_2_O)), and calcium chloride hydroxide (34.0 wt % CaClOH).

**Figure 5:**
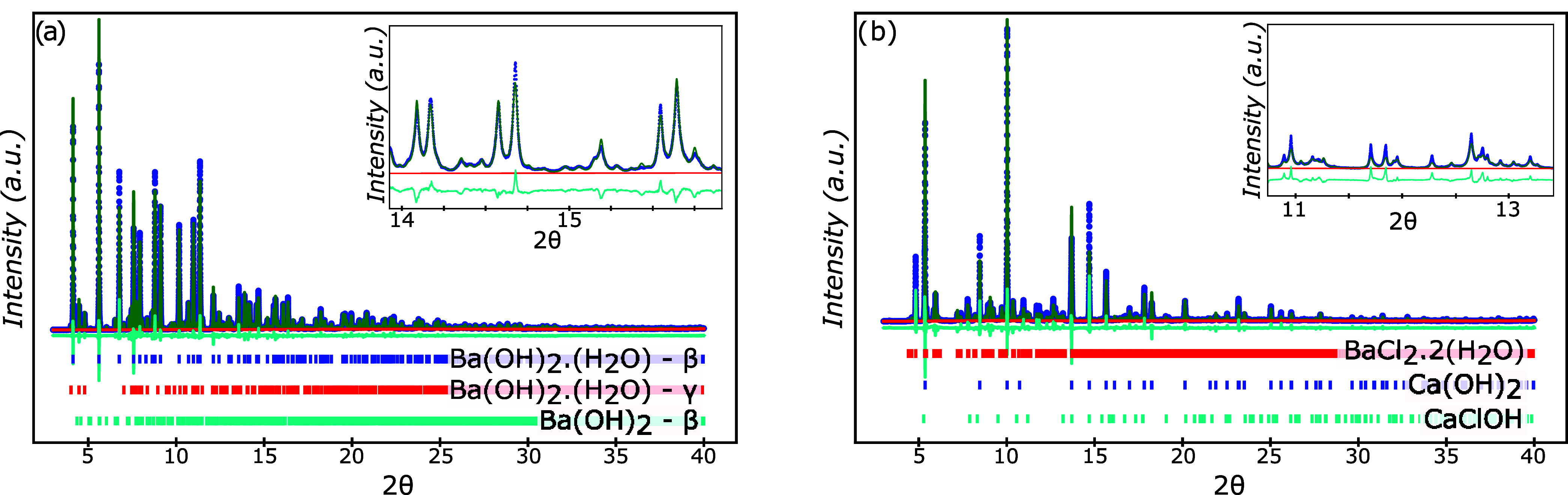
Refined powder diffraction data for (a) as-received barium oxide (BaO), that matches well to the calculated powder diffraction patterns for β-barium hydroxide monohydrate (77.0 wt % β-Ba(OH)_2_·(H_2_O)), γ-barium hydroxide monohydrate (12.6 wt % γ-Ba(OH)_2_·(H_2_O)), and β-barium hydroxide (10.4 wt % β-Ba(OH)_2_), and (b) reacted barium oxide (BaO), that matches well to the calculated powder diffraction patterns for calcium hydroxide (71.6 wt % Ca(OH)_2_), barium chloride dihydrate (24.4 wt % BaCl_2_·2(H_2_O)), and calcium chloride hydroxide (4.0 wt % CaClOH).

**Figure 6:**
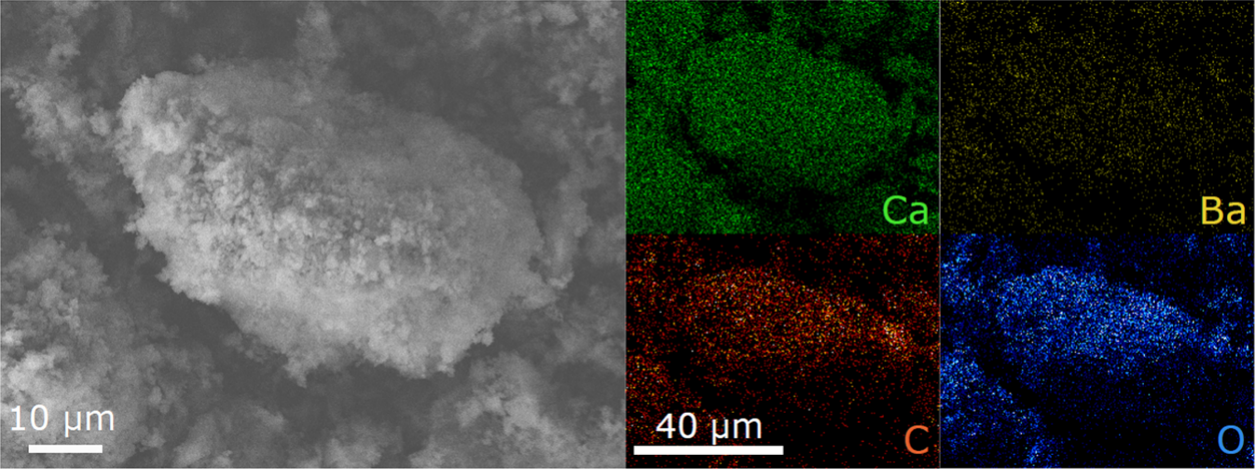
EDS maps depicting a reacted barium carbonate (BaCO_3_) particle. Compositional maps indicate some degree of residual Ba present in the particle. All images correspond to the scale bars in the left-hand corners of the image.

After equilibrating in CCH, a cation substitution reaction was observed to occur in a number of precursor barium compounds (*e*.*g*., BaCO_3_, Ba(OH)_2_), but it does not appear to be primarily responsible for the efficacy of these NPs. To evaluate potential chemical reactions (eqs 1 and 2), the residual solid NPs were separated from the liquid, were rinsed, and then dried (which will henceforth be referred to as “reacted NPs”). XRD analysis on the reacted NPs revealed the reaction of the initial Ba carbonate or hydroxide phase to a dominant calcium carbonate phase (Figures 3 and 4) and calcium hydroxide phase (Figures 5 and 6), respectively.
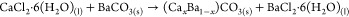
1
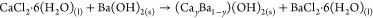
2

These reactions are consistent with changes in melting behavior upon adding greater concentrations of solid Ba-based NPs described before (Figure 2).

Despite being present in the reacted nucleation particles as main products of the reaction between CCH and the NPs, pure calcium hydroxide and calcium carbonate were each evaluated individually and were observed to be ineffective as nucleation particles for CCH. Chemically pure calcium hydroxide and calcium carbonate were each added directly (in isolation) to pure CCH but were observed to be ineffective as nucleation particles for CCH. In contrast, the reacted nucleation particles, which had been separated, rinsed, and dried, were subsequently recombined with pure CCH, and retained their efficacy as nucleators of CCH. This suggests that, despite the observation of some extent of cation exchange reaction, chemically pure calcium-containing phases in isolation are not active nucleants for solid CCH.

### 3.5 Secondary Precipitate Reactions

Based on synchrotron X-ray diffraction data, multiple minor secondary phases were observed in dried reacted NPs. The as-received BaCO_3_ precursor was identified as phase-pure barium carbonate, while the reacted BaCO_3_ was observed to contain a number of phases, including the primary CaCO_3_ (calcite) phase, as well as barium chloride dihydrate, BaCl_2_·2(H_2_O), barium chloride monohydrate, BaCl_2_·(H_2_O), and at least one unresolved minor phase (Figure 3). No higher hydrates of barium chloride have been previously reported under ambient pressure conditions.^41^Equation 3 shows a modified chemical reaction that occurs when solid BaCO_3_ particles are introduced into liquid CCH
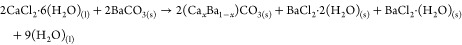
3

Figure 4 shows the as-received and reacted barium hydroxide, with the as-received sample identified as a mixture of two different barium hydroxide-based hydrates. For the reacted samples, the peaks that were not associated with calcium hydroxide were identified as barium chloride dihydrate, (BaCl_2_·2(H_2_O)), barium chloride monohydrate, (BaCl_2_·(H_2_O)), calcium chloride hydroxide, (CaClOH), and another unidentified phase. The as-received and reacted barium oxide samples can be seen in Figure 5, where the as-received sample also contained several different barium hydroxide hydrates. This observation is consistent with previous observations that barium oxide reacts with water vapor to form barium hydroxide and related hydrates. The reacted barium oxide and barium hydroxide were composed of an identical phase assemblage, which is consistent with the fact that both Ba(OH)_2_ and BaO will react with water in the environment to form Ba(OH)_2_-based hydrates. Equation 4 shows the proposed chemical reaction that occurs while the Ba(OH)_2_ nucleation particle is in solution with the calcium chloride hexahydrate. For barium hydroxide, the initial phases observed were β–barium hydroxide monohydrate (Ba(OH)_2_·H_2_O), barium hydroxide trihydrate (Ba(OH)_2_·3H_2_O), and barium peroxide (BaO_(2−δ)_), and the final phases observed were (after reaction and isolation from solution) calcium hydroxide (Ca(OH)_2_), barium chloride dihydrate (BaCl_2_·2(H_2_O)), and calcium chloride hydroxide (CaClOH). Equation 5 shows the proposed chemical reaction that occurs while the BaO nucleation particle is in solution with the calcium chloride hexahydrate. For barium oxide, initial phases included β–barium hydroxide monohydrate (β-Ba(OH)_2_·H_2_O), γ–barium hydroxide monohydrate (γ–Ba(OH)_2_·H_2_O), and β–barium hydroxide (β-Ba(OH)_2_) were observed, and the final phases observed (after reaction and isolation from solution) were calcium hydroxide (Ca(OH)_2_), barium chloride dihydrate (BaCl_2_·2(H_2_O)), and calcium chloride hydroxide (CaClOH). Table 4 displays a summary of all the initial and reacted phases for all the tested samples. Tables 5, 6, and 7 display the crystallographic information for the GSAS-II refinements of the reacted barium carbonate, barium hydroxide, and barium oxide.
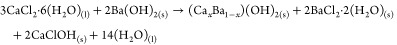
4

**Table 4:** Phase Fraction Composition of Reacted Samples

		(Ca_*x*_Ba_1–*x*_)CO_3_ (wt %)	(Ca_*y*_Ba_1–*y*_)(OH)_2_ (wt %)	BaCl_2_·2(H_2_O) (wt %)	CaClOH (wt %)	BaCl_2_·(H_2_O) (wt %)
nominal initial composition	BaCO_3_	94.7		3.8		1.1
	Ba(OH)_2_		36.3	29.7	34.0	
	BaO		71.6	24.4	4.0	

**Table 5:** Crystallographic Information for GSAS-II Refined Phases in Reacted Barium Carbonate

	(Ca_*x*_Ba_1–*x*_)CO_3_	BaCl_2_·2(H_2_O)	BaCl_2_·(H_2_O)
space group	*R*3̅*c* (No.167)	*P*2_1_/*n* (No.14)	*Pnma* (No.62)
*a*/Å	4.9881 (4)	6.7221 (14)	11.0891 (6)
*b*/Å	4.9881 (4)	10.9098 (19)	4.4927 (3)
*c*/Å	17.1383 (9)	7.1310 (14)	9.0473 (7)
β/°	90	91.093	90
*Z*	6	4	4

**Table 6:** Crystallographic Information for GSAS-II Refined Phases in Reacted Barium Hydroxide

	(Ca_*y*_Ba_1–*y*_)(OH)_2_	BaCl_2_·2(H_2_O)	CaClOH
space group	*P*3̅*m*1 (No.164)	*P*2_1_/*n* (No.14)	*P*6_3_*mc* (No. 186)
*a*/Å	3.5861 (19)	6.7077 (4)	3.8534 (9)
*b*/Å	3.5861 (19)	10.8872 (7)	3.8534 (9)
*c*/Å	4.9013 (18)	7.1162 (5)	9.88995 (18)
β/°	90	91.096	90
*Z*	1	4	2

**Table 7:** Crystallographic Information for GSAS-II Refined Phases in Reacted Barium Oxide

	(Ca_*y*_Ba_1–*y*_)(OH)_2_	BaCl_2_·2(H_2_O)	CaClOH
space group	*P*3̅*m*1 (No.164)	*P*2_1_/*n* (No.14)	*P*6_3_*mc* (No. 186)
*a*/Å	3.5918 (30)	6.7188 (15)	3.8503 (10)
*b*/Å	3.5918 (30)	10.9041 (24)	3.8503 (10)
*c*/Å	4.9101 (30)	7.1268 (16)	9.9798 (27)
β/°	90	91.081	90
*Z*	1	4	2


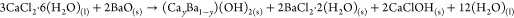
5

Despite the dominant phase adopting the calcium-containing structure in each case (CaCO_3_, Ca(OH)_2_), chemical and structural evidence supports the existence of a limited solid solution within this dominant phase. No intermediate double carbonates (*e*.*g*., BaCa(CO_3_)_2_) were observed.^42^ EDS mapping suggested the presence of residual Ba in the carbonate and hydroxide phases, despite these phases adopting the CaCO_3_ and Ca(OH)_2_ crystal structures (Figure 6). Additionally, resolved lattice parameters in cation-substituted phases were somewhat larger than their pure Ca-containing equivalents (*e*.*g*., the *c* lattice parameter of the resulting (Ca_*x*_Ba_1–*x*_)CO_3_ was resolved to be 17.1383(9) whereas the *c* lattice parameter of pure CaCO_3_ was resolved to be 17.07).^36^ While the chemical composition of this phase was not determined in this study, prior investigation suggests a maximum solubility of Ba cations in CaCO_3_ of (Ba_0.25_Ca_0.75_)CO_3_.^43^ To our knowledge, the degree of solubility of Ba in Ca(OH)_2_ has not previously been reported.

In all cases possible, secondary phases that were identified in the reacted nucleation particles were isolated and tested individually as nucleation particles for calcium chloride hexahydrate. However, none of the secondary phases tested (including BaCl_2_·2(H_2_O), Ca(OH)_2_, CaCO_3_) were effective individually in their chemically pure form as nucleation particles, having little effect on the undercooling in calcium chloride hexahydrate. However, as reported initially, the increased nucleation activity was positively associated with the solid residue that remained after the system reacted. Thus, we infer that the tendency for undercooling to decrease in the presences of different Ba-containing compounds implies either: (1) trace amounts of one or more additional phases were present which were not resolved in synchrotron XRD experiments, or (2) some limited solid-solution was exhibited in the residual solid phases, which resulted in an increased chemical tendency of these precipitates to nucleate crystalline calcium chloride hexahydrate.

While all of the major peaks in the reacted samples’ spectra were identified (Figures 3–5), there were still a few minor peaks that remained unidentified and did not match any previously reported powder diffraction patterns, given the compositional constraints of the system. However, the positions of the unidentified peaks varied from sample to sample, and thus it is unlikely that there is a singular unidentified phase that is responsible for the reduction in undercooling of the CCH.

Additionally, as discussed previously, it appears that a limited extent of barium–calcium solid solution is present within the compound that comprises the largest weight fraction of the solid residual particles. While the exact mechanism by which these solid solution particles may enhance nucleation is unclear, we hypothesize that the high concentration of atomic scale point defects and defect clusters exposed on the surface of the solid solution particles may increase nucleation rates, as similar defect-modulated nucleation behavior has been previously reported from either point defects or from other atomic-scale defects.^44,45^ Regardless, the existence of a majority solid phase with substantial solid solution directly results from in situ reactions between CCH solution and initial starting template solids, introducing a potential vector for developing similar low-undercooling salt hydrate systems in the future.

## 4 Conclusions

Nucleation particles are solid phases that are added to a system to lower the energy required for the system to solidify or undergo a phase transition. Usually, an effective nucleation particle is chosen based on its structural or interfacial energy interaction with the host phase, but this method is premised upon the idea that the nucleation particle is nonreactive with the host phase. In this study, we demonstrate the occurrence of chemical reactions between precursor compounds and a reactive salt hydrate host phase change material, which generate solid products that dramatically reduce undercooling in the host phase change material. While this reactivity complicates the identification of a single phase which is primarily responsible for the nucleation activity, it also introduces a novel approach to harness in situ reactions to generate active nucleation sites within a phase change material. We hypothesize that the efficacy of barium-based nucleation particles in reducing undercooling in CCH is potentially due not to underlying lattice or structural relationships, but rather to the existence of atomic-scale defects on the surface of a solid solution phase. Thus, under this hypothesis, the reactivity between the PCM (CCH) and the original NP is essential to generate a heterogeneous solid template which is observed to be active as a nucleation surface. This strategy potentially introduces a more general approach to identifying NPs for reactive PCMs, including salt hydrates.
